# Application of Behavior Change Techniques (BCTTv1) to Reduce Antimicrobial Use in Livestock: A Scoping Review

**DOI:** 10.3390/vetsci12020172

**Published:** 2025-02-14

**Authors:** Ruth Omani, Folorunso O. Fasina, Peter Kimeli, Nicolas Antoine-Moussiaux

**Affiliations:** 1 Department of Veterinary Management of Animal Resources, Faculty of Veterinary Medicine, University of Liège, 4000 Liège, Belgium; 2 Emergency Center for Transboundary Animal Diseases (ECTAD), Food and Agriculture Organization of the United Nations, Nairobi 00100, Kenya; 3 Food and Agriculture Organization of the United Nations, 00153 Rome, Italy; 4 Department of Veterinary Tropical Diseases, Faculty of Veterinary Science, University of Pretoria, Onderstepoort 0110, South Africa; 5 Department of Clinical Studies, Faculty of Veterinary Medicine, University of Nairobi, Nairobi 00625, Kenya

**Keywords:** antimicrobial use, behavior change techniques, BCTTv1, livestock, antimicrobial resistance, scoping review

## Abstract

Antimicrobial resistance (AMR) is a critical global health threat, partly driven by the overuse of antimicrobials in livestock production. Addressing this issue requires targeted interventions to influence behavioral drivers behind AMU practices. Behavior change techniques (BCTs) offer a structured approach to designing such interventions, yet their application in reducing AMU on farms remains underexplored. This scoping review systematically maps the current applications of the Behavior Change Techniques Taxonomy version 1 (BCTTv1) in interventions aimed at reducing AMU in livestock, while also identifying gaps in the literature and opportunities for future research. A comprehensive search across academic databases identified 23 relevant studies. Findings revealed the use of 37 BCTs, with commonly applied techniques including goal setting, instruction on behavior, credible sources, problem-solving, feedback on behavior, and self-monitoring. While some studies explicitly referenced BCTs, many applied them implicitly, without alignment with standardized frameworks. Intervention outcomes were influenced by contextual factors including financial, logistical, cultural, and technical challenges. This review underscores the importance of explicitly integrating and systematically reporting BCTs to enhance transparency, replicability, and scalability. Future interventions should adopt tailored, context-specific approaches to address regional and sectoral challenges, ultimately contributing to global efforts in combating AMR through improved antimicrobial stewardship.

## Simple Summary

This research explores how behavior change techniques can help reduce antibiotic use in farm animals. The overuse of antibiotics in livestock contributes to antimicrobial resistance, making infections harder to treat in both animals and humans. The study reviews how these techniques have been used to encourage farmers to use fewer antibiotics and identifies areas in which more research is needed. Some effective approaches include setting goals, learning from experts, problem-solving, receiving feedback, and self-monitoring. However, the success of these techniques depends on factors like cost, farm conditions, cultural practices, and education. Many studies do not clearly describe how they apply these techniques, limiting opportunities to refine and replicate successful interventions. To improve antibiotic reduction efforts, future programs should focus on clear documentation, long-term support, and region-specific solutions. Collaboration between veterinarians, farmers, researchers, and policymakers is key to ensuring lasting improvements. More attention is needed in developing countries where antibiotic reduction efforts are limited. Encouraging responsible antibiotic use through behavior change techniques will help slow resistance, assure food safety, protect public health, and improve animal welfare worldwide.

## 1. Introduction

Antimicrobial resistance (AMR) poses significant risks to both food safety and zoonotic disease control. The overuse and misuse of antimicrobials in livestock production are key drivers of AMR, contributing to the emergence and spread of resistant pathogens across animal, human, and environmental ecosystems [1,2]. Despite global and national action plans aimed at mitigating AMR, antimicrobial use (AMU) in livestock remains high, especially in regions with limited regulatory oversight, inadequate veterinary services, and socioeconomic barriers [3,4]. In low- and middle-income countries (LMICs), these challenges are compounded by weak enforcement of regulations and reliance on antimicrobials for both prophylactic and therapeutic purposes [4,5]. The Global Action Plan (GAP) on AMR emphasizes the importance of reducing antimicrobial dependence in agriculture through sustainable practices and effective behavioral interventions [6]. However, successful implementation of these strategies requires an understanding of the behavioral, economic, and structural factors driving AMU on farms [7,8]. Farmers and animal health service providers often face challenges such as financial pressures, limited technical knowledge, and deeply rooted cultural norms that hinder the adoption of antimicrobial stewardship practices [8,9,10].

Behavior change techniques (BCTs) have emerged as essential tools for designing and implementing interventions aimed at changing human behavior [11,12,13,14]. BCTs refer to identifiable, replicable components of behavior change interventions designed to modify causal processes underlying behavior [11,12,13,14]. The Behavior Change Techniques Taxonomy version 1 (BCTTv1) provides a standardized framework comprising 93 techniques across 16 categories, offering a structured approach to developing and evaluating behavior change interventions [11,12,13,14]. While evidence suggests that behavior change techniques (BCTs) have been effective in driving behavior change in other sectors, including in the healthcare settings [13,15,16,17,18], their application in agricultural settings, particularly in reducing antimicrobial use (AMU) on farms, remains relatively underexplored. This gap underscores the need to better understand the behavioral, economic, and cultural drivers that influence AMU practices among farmers and animal health service providers, especially in low- and middle-income countries (LMICs).

This study aimed to map the application of BCTs in interventions designed to reduce on-farm AMU, identifying commonly used techniques, their effectiveness, and the contextual factors influencing their success. By systematically reviewing the existing literature, this study seeks to provide evidence-based insights into how BCTs can be effectively integrated into livestock interventions to drive sustainable behavior change and reduce reliance on antimicrobials. The findings aim to bridge knowledge gaps, inform policy, and guide future intervention design, contributing to safer food systems and mitigating zoonotic risks.

## 2. Materials and Methods

### 2.1. Study Design and Research Questions

This scoping review followed the Arksey and O’Malley framework and adhered to the PRISMA-ScR (Preferred Reporting Items for Systematic Reviews and Meta-Analyses Extension for Scoping Reviews) checklist for systematic reporting. This approach was deemed suitable for mapping and synthesizing evidence related to the application of behavior change techniques (BCTs) in interventions aimed at reducing on-farm antimicrobial use (AMU) [19,20]. Scoping reviews are particularly useful for exploring complex, interdisciplinary topics like BCTs, where existing evidence is diverse and spans multiple contexts, allowing for a comprehensive understanding of current practices, gaps, and opportunities for future research [20]. This review complied fully with PRISMA-ScR guidelines to ensure transparency, rigor, and reproducibility.

The research question was formulated using the PICO (population, intervention, comparator, and outcome) framework to ensure clarity and focus (See Table 1 below).

### 2.2. Search Strategy, Eligibility Criteria, and Screening Process

A search strategy was employed to identify relevant literature addressing the use of behavior change techniques (BCTs) in interventions aimed at reducing on-farm antimicrobial use (AMU). The search was conducted across multiple electronic databases, including PubMed, Web of Science, Scopus, and Google Scholar, on 13 December 2023. The search terms combined keywords and Boolean operators such as “antimicrobial use”, “behavior change technique”, and “livestock”, with variations like “antibiotic use” and “husbandry”. An example search string included the following: ((“antimicrobial use” OR “antimicrobial usage” OR “antibiotic use” OR “antibiotic usage” OR, “antimicrobial resistance” OR “antibiotic resistance OR “AMU” OR “AMR”) AND (intervention OR program OR project OR planning OR plan OR initiative OR action OR strategy OR policy OR guideline OR regulation OR legislation OR control OR monitoring OR stewardship OR “behavior change technique” OR “behavior change” OR “BCT”) AND (veterinary OR farm OR livestock OR husbandry OR breeding OR herd OR animal OR poultry OR chicken OR broiler OR hen OR duck OR cattle OR cow OR calves OR veal OR beef OR dairy OR pig OR swine OR pork OR hog OR sow OR piglet OR sheep OR goat)). No restrictions on publication date or geographic location were applied.

The inclusion criteria focused on studies that explicitly or implicitly reported the use of BCTs in interventions to reduce AMU among livestock farmers or animal health providers. Eligible studies included peer-reviewed articles, reports, and theses incorporating quantitative, qualitative, or mixed-method approaches. Studies were excluded if they did not focus on AMU in livestock, were review articles without original data, or were limited to conference abstracts and opinion pieces.

The screening process was conducted in two stages. First, the titles and abstracts of the retrieved studies were independently reviewed by two researchers to eliminate irrelevant records. In the second stage, full-text articles of potentially eligible studies were assessed against the inclusion and exclusion criteria. Discrepancies in study selection were resolved through consensus, with a third reviewer consulted when necessary. Rayyan QCRI software (2023) was utilized to streamline the screening and selection process, ensuring consistency and reducing reviewer bias. This approach ensured a systematic and transparent identification of relevant literature, aligning with the scoping review’s objective to map the application of BCTs in interventions aimed at reducing antimicrobial use on farms.

### 2.3. Data Synthesis and Quality Assessment

The data extracted from the included studies were analyzed using a narrative synthesis approach, allowing for the identification and mapping of behavior change techniques (BCTs) applied in interventions aimed at reducing on-farm antimicrobial use (AMU). Data extraction focused on study characteristics, intervention descriptions, types of BCTs used (both explicitly and implicitly), outcomes achieved, and contextual factors such as barriers and enablers.

To ensure alignment with the study objectives, the synthesis was organized around key themes, including the identification of BCTs, their explicit vs. implicit application, their effectiveness across intervention strategies (e.g., herd management, antimicrobial stewardship training, incentives, regulations, and targeted treatments), and their geographic and sectoral distribution. These themes provided a structured lens through which patterns and trends were examined.

While quality assessment is not a requirement for scoping reviews, a risk of bias assessment was performed using the Mixed Methods Appraisal Tool (MMAT). This ensured a baseline evaluation of the methodological rigor and reliability of the included studies, contributing to a more transparent and credible synthesis of the findings.

### 2.4. Selection and Characteristics of Sources of Evidence

**Figure 1:**
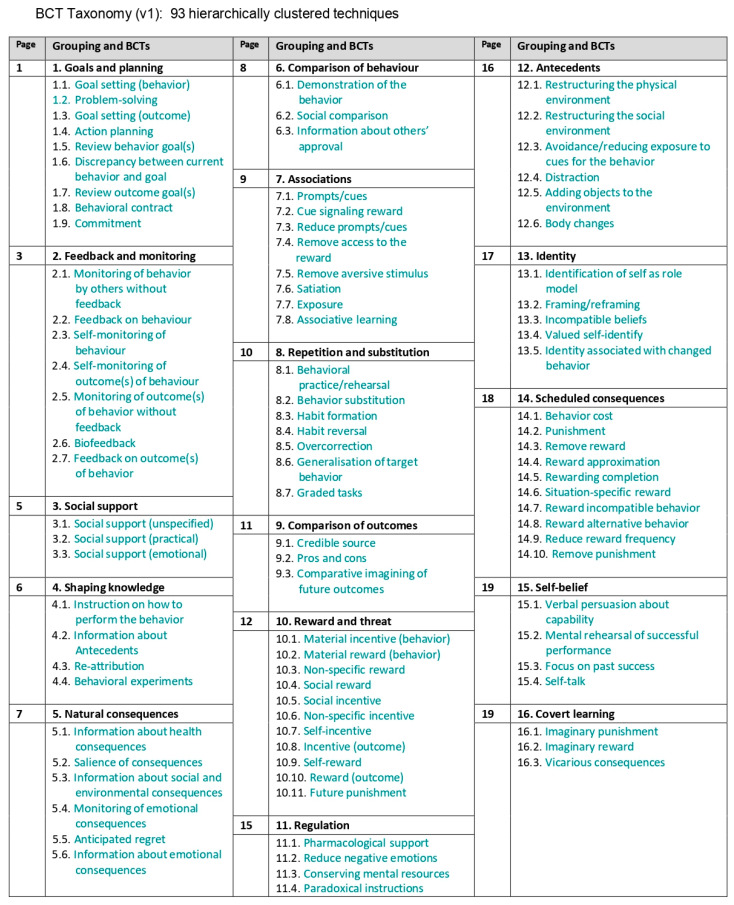
Overview of the Behavior Change Techniques (BCT) Taxonomy (v1), illustrating the 93 hierarchically clustered techniques categorized into 16 groups [13].

**Figure 2:**
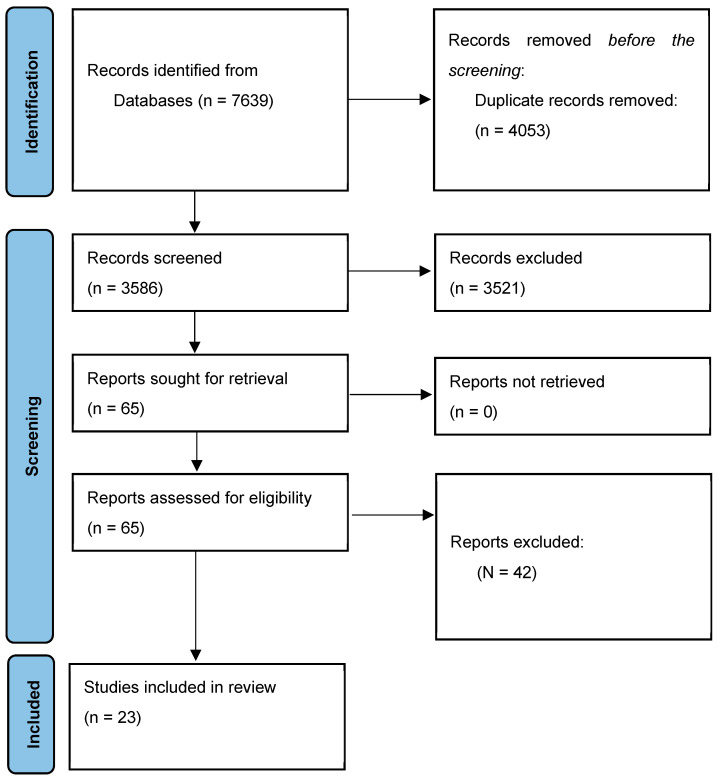
PRISMA flow diagram showing the selection process for studies included in the scoping review.

A comprehensive search yielded 7639 records, which were screened to remove 4053 duplicates, resulting in 3586 unique studies. These studies underwent a two-stage relevance assessment conducted independently by two researchers to ensure objectivity and minimize bias. The initial screening criteria focused on identifying studies that examined on-farm interventions designed to reduce antimicrobial use (AMU). Through this rigorous process, 65 studies were identified for full-text review (see Figure 1). During the full-text evaluation, studies were excluded if they lacked sufficient detail on interventions, were unrelated to AMU in livestock, or did not explicitly or implicitly involve BCTs. This led to the selection of 23 studies that met the inclusion criteria and were subjected to a detailed analysis of BCTs using the Behavior Change Techniques Taxonomy version 1 (BCTTv1) (see Figure 2). To enhance the validity and reliability of BCT coding, the reviewers engaged in training sessions using the BCT Taxonomy Training website (https://www.bct-taxonomy.com/, accessed on 6 January 2024), ensuring consistent and accurate application of the BCTTv1 framework.

## 3. Results

### 3.1. Identification of Behavior Change Techniques (BCTs) Used in Interventions to Reduce AMU

**Figure 3:**
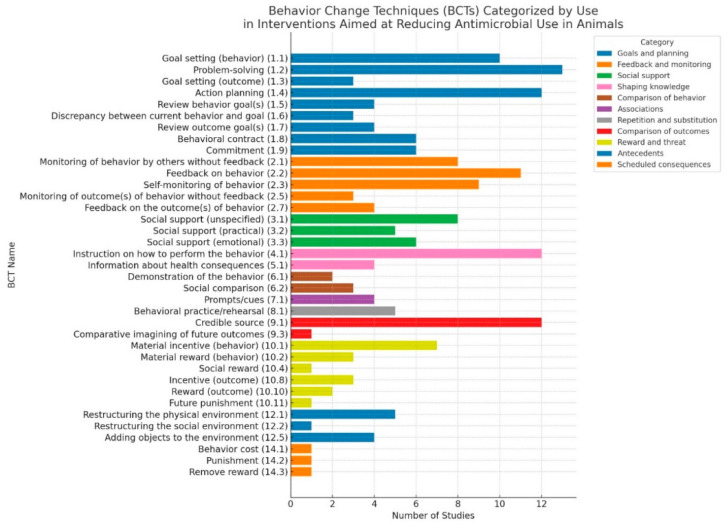
Behavior change techniques (BCTs) are categorized by their use in interventions to reduce antimicrobial use (AMU) in animals.

This scoping review identified 37 of the 93 BCTs outlined in the BCT Taxonomy version 1 (BCTTv1) across 23 studies (See Supplementary Material Table S1). These techniques were used explicitly and implicitly in interventions to reduce AMU in livestock production systems. The BCTs were categorized into several groups, including goals and planning, feedback and monitoring, social support, shaping knowledge, and others (see Figure 3 below). The most frequently used BCTs were problem-solving [21,22,23,24,25,26,27,28,29,30,31,32], instruction on how to perform the behavior [21,23,26,28,30,32,33,34,35,36,37,38] relying on credible sources [23,24,27,29,30,31,33,34,35,37,38,39], action planning [21,22,23,25,27,29,31,33,34,38,40,41], feedback on behavior [21,23,25,26,27,29,31,36,37,39,41], goal setting [21,23,25,26,27,29,31,36,37,39,41], and self-monitoring of behavior [22,26,32,33,34,35,39,41,42]. These were cited seven to thirteen times across the 23 publications (See Supplementary Material Table S1).

### 3.2. Explicit vs. Implicit Use of BCTs in Interventions

Of the 23 studies reviewed, only 7 explicitly mentioned BCTs or used taxonomy-related behavioral terms. The remaining 16 studies applied BCTs implicitly, often embedding them within broader intervention frameworks such as training programs, incentive systems, or policy changes, without explicitly labeling them as BCTs. The seven studies that specifically mentioned behavior change techniques (BCTs) emphasized the effective incorporation of social and behavioral techniques into interventions to reduce on-farm AMU. These studies illustrated that by addressing the behavioral factors that influence AMU, it is possible to achieve sustained AMU reduction [23,29,36,37,38,41,43]. The 16 studies did not expressly mention BCTs primarily. Although these interventions led to a reduction in AMU, they overlooked the chance to address the social and behavioral dimensions of AMU. For example, studies that examined the effects of regulatory measures on AMU [25,32], while providing valuable evidence of the effectiveness of these measures, did not investigate how these regulations affected the behaviors and attitudes of farmers.

### 3.3. Effectiveness of BCTs Across Intervention Strategies

The effectiveness of behavior change techniques (BCTs) in interventions aimed at reducing antimicrobial use (AMU) on farms was assessed across five key intervention strategies: Optimization of Herd Management, Antimicrobial Stewardship Training, Incentives-Based Systems, Regulatory Frameworks, Targeted Treatments, and Alternative Approaches. Each strategy demonstrated varying degrees of success, influenced by contextual factors such as infrastructure, financial resources, regulatory support, and stakeholder engagement. The summary of these findings is presented below (see Table 2).

### 3.4. Geographic and Sectoral Distribution of Interventions

**Figure 4:**
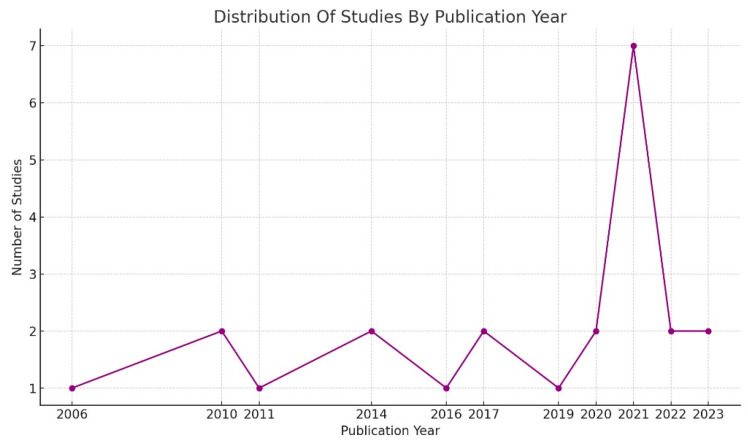
Distribution of studies by publication year.

The scoping review included research papers published from 2006 to 2023 (see Figure 4), with interventions implemented in the bovine (47.8%), swine (34.8%), and poultry (17.4%) sectors. Most interventions (16) were implemented in European countries, followed by North America (4), Asia (2), and Africa (1). All interventions targeted farmers, with only three publications noting animal health service providers as a focus. The most used research methods were observational, longitudinal, and quasi-experimental designs.

### 3.5. Key Barriers and Enablers to Effective Implementation of BCTs

Barriers and enablers that influenced interventions to reduce antimicrobial use (AMU) in livestock were categorized into financial, logistical, cultural, behavioral, and technical themes (see Table 3 below). Financial barriers included the high costs of veterinary services and diagnostics, while incentives and subsidies acted as enablers. Logistical challenges arose from inconsistent follow-ups and limited monitoring tools. Cultural barriers involved entrenched traditions and reliance on prophylactic AMU. Behavioral enablers, such as self-monitoring tools, fostered accountability, while technical barriers highlighted gaps in advisory services. Veterinarians and peer leaders served as trusted enablers for intervention success. Addressing these factors was critical for designing sustainable and effective AMU reduction strategies.

## 4. Discussion

The findings of this scoping review provide a comprehensive understanding of how behavior change techniques (BCTs) have been integrated into interventions aimed at reducing on-farm antimicrobial use (AMU). A total of 37 distinct BCTs, derived from the Behavior Change Techniques Taxonomy version 1 (BCTTv1), were identified across 23 included studies. However, a striking observation was the inconsistency in the explicit reporting and integration of these techniques into intervention designs. Only seven studies explicitly referenced BCTs, aligning their methodologies with recognized taxonomies. In contrast, the majority implicitly applied behavioral principles, without systematically identifying or documenting them. This inconsistency highlights a significant gap in the field, where technical, regulatory, or economic priorities often overshadow behavioral dimensions [46,47,48]. The explicit integration of behavior change techniques (BCTs) is essential for enhancing transparency, ensuring replicability, enabling scalability, and supporting evidence-based evaluation of interventions across diverse contexts [11,13,49,50,51]. Studies that have utilized BCTs provide a more coherent articulation of intervention objectives, behavioral mechanisms, and anticipated outcomes. In contrast, while still demonstrating effectiveness, implicit applications often lack clarity regarding behavioral strategy selection, deployment, and measurement. Future research and intervention design should prioritize the thorough documentation of BCTs using standardized taxonomies like BCTTv1, allowing stakeholders to better understand, replicate, and refine these methodologies.

The most applied BCTs—goal setting, instruction on how to perform behavior, credible sources, problem-solving, feedback on behavior, and self-monitoring—formed the backbone of many reviewed interventions. Goal setting provided farmers with clear, measurable targets for antimicrobial use reduction, often tailored to farm-specific needs [21,23,25,26,27,29,31,36,37,39,41]. Instruction on how to perform behavior offered step-by-step guidance on antimicrobial administration and infection prevention typically delivered through workshops and veterinarian-led demonstrations [21,23,26,28,30,32,33,34,35,36,37,38]. Credible sources, such as veterinarians and agricultural extension workers, played a key role in building trust and delivering reliable advice [23,24,27,29,30,31,33,34,35,37,38,39]. Problem solving, often paired with participatory approaches, empowered farmers to identify and address farm-specific barriers to stewardship using tools like Biocheck.UGent for structured assessments and tailored action plans [21,22,23,24,25,26,27,28,29,30,31,32]. Feedback on behavior reinforced accountability through farm visits, comparative performance reviews, and constructive discussions [21,23,25,26,27,29,31,36,37,39,41]. Meanwhile, self-monitoring allowed farmers to track their antimicrobial use and other farm practices using tools like treatment logs and digital monitoring systems, fostering ownership and precision in AMU practices [22,26,32,33,34,35,39,41,42]. Despite their promise, these techniques often faced financial, logistical, and follow-up challenges, limiting their long-term sustainability. Successful interventions strategically combined multiple BCTs, leveraging their complementary strengths to drive sustained behavior change and improve antimicrobial stewardship outcomes.

Building on the analysis of intervention strategies, it becomes evident that the interaction between behavior change techniques (BCTs) and intervention delivery mechanisms played a decisive role in shaping outcomes. In the Optimization of Herd Management, participatory planning, action planning, and problem-solving enabled farmers to identify herd-specific risks and devise actionable mitigation strategies [22,27,30,31,40]; tools like Biocheck.UGent facilitated structured assessments, leading to measurable improvements in biosecurity practice. However, financial barriers and inconsistent veterinary follow-ups limited scalability, especially in resource-constrained settings [21,30]. Antimicrobial Stewardship Training relied on instruction on performing behavior, credible sources, and self-monitoring to improve AMU practices [21,25,27,28,34,35]. Veterinarians served as trusted information sources, delivering workshops and on-farm demonstrations that enhanced knowledge retention and compliance [25,27,28,30]. However, logistical challenges, including fragmented extension services and cultural resistance, weakened the sustainability of these programs [30,45]. Incentive-based systems leveraged material incentives and social rewards to drive participation, showing short-term improvements in AMU compliance [22,24,25,33,37,44]. Financial compensation and subsidies encouraged adoption, but dependency on external funding and opportunistic behaviors among farmers often undermined long-term success [22,24,33,44,45]. Regulatory Frameworks, such as Denmark’s Yellow Card Scheme, applied BCTs like restructuring physical and social environments and behavior costs to enforce compliance [25,31,32,38,43]. Policies that integrated education and financial incentives alongside enforcement proved more effective, whereas punitive measures or perceived unfairness often fostered resistance [22,25,32,44]. Lastly, Targeted Treatments and Alternative Approaches employed instructional guidance, feedback mechanisms, and self-monitoring tools to reduce blanket AMU practices [22,30,35,36,39]. Techniques such as selective dry cow therapy (SDCT) demonstrated success in controlled settings, but skepticism, limited diagnostic tools, and inadequate technical support hindered their scalability [34,35,39]. These findings highlight the importance of integrating financial sustainability, technical capacity, regulatory fairness, and cultural acceptance into intervention designs. Effective AMU reduction strategies require a multidisciplinary approach that aligns behavior change techniques (BCTs) with local contexts, ensuring sustained adoption and meaningful outcomes.

Geographic disparities in antimicrobial use (AMU) interventions reveal significant challenges for the scalability and generalizability of behavior change techniques (BCTs). The majority of the studies originated from Europe, where established financial resources, regulatory systems, and veterinary infrastructure supported structured interventions, such as Denmark’s Yellow Card Scheme [25,29,32,38]. In contrast, regions like Sub-Saharan Africa and parts of Asia remain underrepresented, despite experiencing disproportionately high AMU levels driven by limited veterinary access, weak regulations, and socio-economic barriers [3,30,36]. Interventions in Europe often combined regulatory frameworks with herd management strategies, while North American efforts focused on stewardship training programs and incentive-based systems [25,29,32,38]. In LMICs, emphasis was placed on knowledge dissemination via instructional training, but fragmented veterinary services and financial limitations undermined their effectiveness [3,30,36,44]. At the sectoral level, bovine (47.8%) and swine (34.8%) systems dominated the evidence base, while poultry farming (17.4%), despite its heavy reliance on antimicrobials, received less attention. Poultry production, particularly in LMICs, often operates under weaker regulatory oversight and limited access to diagnostic tools, making effective intervention delivery challenging [3,30,31,34,36]. Moreover, most interventions primarily targeted farmers, with limited focus on the role of veterinarians and supply chain actors in influencing AMU practices [29,34,36,42]. Addressing these geographic and sectoral imbalances will require region-specific intervention designs, stronger veterinary and advisory support, and better integration of regulatory enforcement with behavioral approaches [22,24,31]. Expanding interventions to underrepresented regions and sectors will ensure broader applicability and sustainability of AMU reduction strategies.

In summary, this scoping review underscores the critical role of behavior change techniques (BCTs) in interventions aimed at reducing antimicrobial use (AMU) on farms, with direct implications for food safety and zoonosis prevention. Effective AMU practices help minimize the risk of antimicrobial residues in animal products while curbing the emergence and transmission of resistant zoonotic pathogens. While interventions varied in their application—spanning explicit and implicit uses of BCTs—their effectiveness was shaped by financial, logistical, cultural, and technical factors, as well as geographic and sectoral contexts. The integration of techniques such as goal setting, instruction on behavior, problem-solving, and self-monitoring demonstrated consistent success across multiple strategies, albeit with varying degrees of sustainability. However, the persistent challenges of financial dependency, fragmented advisory systems, and limited technical capacity highlight the need for tailored interventions that align with local realities. Moving forward, there is an urgent need for the intentional and systematic incorporation of behavioral techniques into AMU reduction interventions. This approach requires multidisciplinary collaboration, combining regulatory enforcement, financial incentives, training programs, and participatory engagement to effectively address the multifaceted barriers to AMU reduction. Addressing evidence gaps—particularly in underrepresented regions and sectors, such as poultry farming and LMICs—will be critical for designing scalable and context-specific interventions that can meaningfully tackle the global challenge of antimicrobial resistance.

## 5. Conclusions

This scoping review systematically examined the application of behavior change techniques (BCTs) in interventions aimed at reducing on-farm antimicrobial use (AMU). Across 23 studies, 37 BCTs from the Behavior Change Techniques Taxonomy version 1 (BCTTv1) were identified, with Problem Solving, Instruction on How to Perform the Behavior, Credible Sources, Action Planning, and Feedback on Behavior emerging as the most frequently applied strategies. However, inconsistent identification and documentation of BCTs remain a significant barrier, with only seven studies explicitly referencing BCTs in their design and implementation. This inconsistency reduces transparency, comparability, and the potential for robust evaluation and scalability. Future interventions must prioritize the explicit integration and systematic reporting of BCTs using standardized taxonomies like BCTTv1. Intervention strategies such as herd management optimization, antimicrobial stewardship training, incentive-based systems, regulatory frameworks, and targeted treatments demonstrated varying degrees of success, influenced by financial, logistical, cultural, and technical barriers. While training programs and herd management approaches showed promise through participatory and individualized action plans, incentive-based systems faced sustainability challenges, and regulatory frameworks often encountered resistance due to high compliance costs and mistrust. Geographic and sectoral disparities persist, with most studies originating from Europe and North America, while regions like Asia and Africa and sectors such as poultry farming remain underrepresented. Addressing these imbalances will require context-specific interventions and targeted research efforts.

To enhance the effectiveness of BCT-based interventions in reducing AMU in the livestock sector, future efforts should be grounded in interdisciplinary collaboration, engaging experts across public health, veterinary medicine, behavioral science, and agricultural sectors including international agencies, research institutions, and professional networks. These collaborations should foster knowledge exchange, practical interventions, robust evaluation frameworks, and capacity-building initiatives in the application of BCTs to drive sustainable antimicrobial stewardship solutions in livestock systems. The following priorities should guide these efforts:

- Explicit integration and systematic reporting of BCTs using taxonomies like BCTTv1 to enhance transparency, replicability, and comparability across interventions, enabling more effective evidence synthesis.
- Context-specific intervention designs adaptable to regional, cultural, and economic settings to ensure feasibility, relevance, and a higher likelihood of adoption among key stakeholders.
- Focused efforts in underrepresented regions and sectors, particularly Asia, Africa, and poultry farming to address existing gaps, ensure the equitable distribution of resources, and maximize the global impact of AMU reduction strategies.
- Sustainability mechanisms, including financial and structural models for long-term viability to support continued implementation beyond the initial phases, ensuring that improvements in AMU practices are sustained over time.
- Robust monitoring and evaluation frameworks to track long-term impacts and refine approaches to enable continuous assessment, facilitate early identification of challenges, and support the iterative adaptation of interventions.

In summary, behavior change techniques (BCTs) are a powerful yet underutilized tool in antimicrobial stewardship interventions. Their integration into technical, policy-driven, and behavioral strategies offers a pathway toward sustainable reductions in antimicrobial use. Moving forward, collaborative, multidisciplinary, and context-aware approaches will be essential to overcoming systemic barriers and embedding antimicrobial stewardship as a sustainable practice in global livestock production systems.

## Figures and Tables

**Table 1:** PICO framework for evaluating behavior change techniques in reducing on-farm antimicrobial use.

Category	Component
Population	Livestock stakeholders, including farmers and animal health service providers, are involved in the management of farm animals (e.g., cattle, poultry, swine, sheep) who are responsible for the administration of antibiotics.
Intervention	Interventions that utilize behavior change techniques to reduce on-farm AMU.
Comparator	Interventions that do not use behavioral change techniques to reduce on-farm AMU.
Outcome	On-farm antimicrobial reduction (primary). Other improvements in biosecurity.

**Table 2:** Key intervention strategies for reducing antimicrobial use (AMU) in livestock.

Intervention Strategy	Key BCTs Applied	Example Studies	Challenges	Key Insights
Optimization of Herd Management	Problem Solving, Action Planning, Feedback on Behavior, Goal Setting (Outcome), Monitoring of Outcomes Without Feedback	[21,23,25,29,31,41]	High initial costs, infrastructure limitations, logistical constraints.	Tailored interventions with participatory planning enhance herd management outcomes.
Antimicrobial Stewardship Training	Instruction on How to Perform the Behavior, Self-Monitoring, Credible Sources, Behavioral Practice/Rehearsal	[25,28,29,35,36,44]	Resistance to change, logistical barriers, inconsistency in training delivery.	Combining theoretical training with hands-on practice improves adoption and effectiveness.
Incentives-Based Systems	Material Incentives, Social Rewards, Comparative Imagining of Future Outcomes, Financial Compensation	[24,25,27,30,37]	Sustainability of incentives, bureaucratic complexities, resource dependency.	Financial and material incentives must align with long-term sustainability goals.
Regulatory Frameworks	Restructuring of Social and Physical Environments, Adding Objects to the Environment, Behavior Cost	[22,25,31,32,37]	Compliance costs, distrust in enforcement, unintended consequences (e.g., increased disease incidents).	Integrating education, incentives, and regulatory frameworks fosters compliance.
Targeted Treatments and Alternative Approaches	Instruction on How to Perform Behavior, Feedback on the Outcomes of Behavior, Goal Setting, Comparative Outcomes	[29,30,35,36,39,45]	Skepticism, resource constraints, need for ongoing validation and demonstration.	Regular technical support and trust-building measures are essential for sustained adoption.

**Table 3:** Barriers and enablers influencing the effectiveness of interventions aimed at reducing antimicrobial use (AMU) in livestock.

Theme	Barrier/Enabler	Key Insights	Examples	Citations
Financial	Barrier	High costs for veterinary services, diagnostics, and infrastructure hinder adoption.	Costs of training programs, diagnostic tools, and biosecurity infrastructure.	[23,31,42]
	Enabler	Financial incentives and subsidies motivate compliance with stewardship practices.	Annual stipends, free inspections, and equipment subsidies.	[24,27,33,42]
Logistical	Barrier	Inconsistent follow-ups, fragmented extension services, and limited monitoring tools hinder sustainability.	Limited access to diagnostic tools and poor follow-up support.	[21,31,35]
Cultural	Barrier	Entrenched traditions, reliance on prophylactic AMU, and stigma resist change.	Generational reliance on antimicrobials; stigma against compliance.	[22,28,45]
Behavioral	Enabler	Self-monitoring tools and participatory approaches drive accountability and ownership.	Use of treatment logs, mobile apps, and participatory planning.	[28,31,34]
Technical	Barrier	Limited access to trusted experts and inconsistent veterinary services limit adoption.	Gaps in advisory visits and inconsistent peer-led programs.	[29,30,34]
	Enabler	Veterinarians, agricultural officers, and peer leaders serve as trusted sources of guidance.	Peer-based learning sessions and trusted veterinary oversight.	[26,32,37]

## Data Availability

All data supporting the findings of this study are included in this article.

## Supplementary Materials

The following supporting information can be downloaded at: https://www.mdpi.com/article/10.3390/vetsci12020172/s1, Table S1: Summary of behavior change techniques (BCTs) utilized in on-farm antimicrobial reduction interventions, with examples and supporting authors.
