# Supplementary material for: Application of Behavior Change Techniques (BCTTv1) to Reduce Antimicrobial Use in Livestock: A Scoping Review
Source: Vet Sci. 2025 Feb 14;12(2):172. doi: 10.3390/vetsci12020172 (PMC11860433; doi:10.3390/vetsci12020172)
Supplement: Supplementary file 1 [file vetsci-12-00172-s001.zip › vetsci-3424504-supplementary.pdf]

Table S1: Summary of Behavior Change Techniques (BCTs) Utilized in On-Farm Antimicrobial Reduction Interventions with Examples and Supporting Authors

BCT category	BCT Number	BCT Name	Verbatim Example	Other Authors
Goals and Planning<br><br>Definition: Techniques to help individuals set specific targets, identify potential obstacles, and create detailed strategies to accomplish their objectives.	1.1	Goal setting (behavior)	The involved dairy company was interested in investigating the possibilities of establishing a separate product line (niche market) from herds with no use of antibiotics [22]	[22–25,28–30,35,36,44]
	1.2	Problem-solving	It was therefore decided that the main approach was to design individual farm and herd strategies through a participatory process using farmer groups for mutual advice and common learning [22]	[22–29,29–33]
	1.3	Goal setting (outcome)	A *Yellow Card* initially releases an order to reduce antimicrobial use below the threshold within nine months [27]	[24,27,29]
	1.4	Action planning	A multidisciplinary team including infectious disease and large animal internal medicine specialists, epidemiologists, veterinary practitioners, and farmers then participated in the development of general recommendations to adjust management practices for improving calf health and immunity, training of farm staff on calf health evaluation, and development of an antimicrobial drug-use algorithm for treatment of diarrhoeic calves [36]	[22–25,27,29,31,33,35,36,40,41]
	1.5	Review behavior goal(s)	Written follow-up surveys were sent to the dairy producers who completed the initial survey again with a US $5 cash incentive [41]	[25,29,31,41]
	1.6	Discrepancy between current behavior and goal	The veterinarian identified instances where the producer recorded different antibiotic use decisions relative to the treatment protocol and these discrepancies were discussed in an unstructured fashion [30]	[25,29,30]
	1.7	Review outcome goal(s)	A second audit was performed within 1 yr after the given advice to evaluate the changes [25]	[24,25,33,37]
	1.8	Behavioral contract	In Belgium, France, and Germany selected alternatives were consigned on a form the farmer as well as the herd veterinarian were asked to sign to confirm they agreed to implement the defined plan from a certain date [24]	[23,24,26,27,35,37]
	1.9	Commitment	Farmers who participate have to keep cows in a free stall housing system as opposed to tie stalls [44]	[22,24,25,31,37,44]
Feedback and Monitoring<br><br>Definition: Behavioral change techniques (BCTs) that facilitate tracking of progress and pinpointing areas for enhancement, thereby promoting accountability and motivation.	2.1	Monitoring of behavior by others without feedback	Study technicians observed herd personnel performing on-farm culture procedures [37]	[24,26,30–32,35–37]
	2.2	Feedback on behavior	The farmer was provided with feedback on the execution of the action plan and further suggestions were discussed [23]	[23,25,27–29,31,33,38,39,41,42]
	2.3	Self-monitoring of behavior	Each farm provided data on biosecurity measures, disease incidence, and antimicrobial expenditures or detailed treatment records during a period of 12 months preceding and 12 months following the initiation of the peer-to-peer intervention study [43]	[24,28,34–37,41–43]
	2.5	Monitoring of outcome(s) of behavior without feedback	During the first herd visit, information was gathered on herd characteristics, management, and technical performances of the herd [31]	[24,31,37]
	2.7	Feedback on the outcome(s) of behavior	Correct use of the innovations was assessed using behavioral observations in which participants were asked to demonstrate their use of the innovation [32]	[24,31–33]
Social Support<br><br>Definition: The act of aiding and encouraging	3.1	Social support (unspecified)	Each participant in the group of farmers hosted the rest of the group on their farm for the first time. This occurred in sequence until everyone in the group had hosted once [29]	[22,23,25,26,28,29,36,38]

others to help achieve changes in behavior, often fostering a sense of community and shared accountability.	3.2	Social support (practical)	At the start of the project the farmers were instructed which symptoms (including symptoms specifically important for homeopathic diagnostics) they had to pay attention to in the event of illness in order to provide the advisor on the phone with as accurate a picture of the animal as possible [28]	[22,24,28,41,42]
	3.3	Social support (emotional)	Interventions were monitored over one year following the beginning of the intervention; the follow-up included a minimum of two farm visits (i.e. one intermediate and one final visit) and a maximum of six farm visits as well as intermediate phone calls with the farmer and the herd veterinarian [24]	[22,24,24,30,31,35]
**Social Comparison**<br><br>Definition: Techniques that involve demonstrating desired behaviors, facilitating social comparisons, and highlighting the benefits of adopting similar practices.	4.1	Instruction on how to perform the behavior	At the meetings, we provided both verbal instructions and direct demonstrations about correct pasteurization and weight estimation for dosage [32]	[23,25,28,30,32,34–40]
**Natural Consequences**<br><br>Definition: Behavioral change techniques that leverage individuals' understanding of the direct and indirect outcomes of their behavior to motivate change.	5.1	Information about health consequences	During these meetings, we presented public health messages regarding the potential for contaminated milk to cause diseases and Maasai difficulties with calculating livestock weight for proper dosing [32]	[23,25,32,41]
**Social Comparison**<br><br>Definition: Techniques that involve demonstrating desired behaviors, facilitating social comparisons, and highlighting the benefits of adopting similar practices.	6.1	Demonstration of the behavior	Correct use of the innovations was assessed using behavioral observations in which participants were asked to demonstrate their use of the innovation [32]	[29,32]
	6.2	Social comparison	Farmers with herds above the 80th percentile received a letter warning them that they were close to Yellow Card thresholds and Yellow Cards were issued from December 2010 onwards [27]	[27,29,40]
**Association**<br><br>Definition: Behavioral change techniques that establish reminders and triggers to prompt individuals to adopt and maintain new practices.	7.1	Prompts/cues	The farmer was notified that a tour around the farm would form part of the visit [31]	[27,27,28,31]
**Repetition and Substitution**<br><br>Definition: Techniques that involve fostering replication of desired behavior or replacing outdated, undesired behaviors with new, advantageous ones.	8.1	Behavioral practice/rehearsal	Directly following the presentations, a member of the team reinforced the didactic content by working directly with the producer on individual calf health assessments [30]	[29,30,30–32]
**Comparison of Outcomes**<br><br>Definition: Techniques that showcase the advantages of desired actions and contrast these results with those of current practices.	9.1	Credible source	Farm staff involved in calf care or directing treatment were trained in health evaluation (recognition of changes in attitude, feed intake, and temperature), use of the algorithm, and administration of intravenous and oral fluids [36]	[24,25,28,30–32,34–36,36,38,39,41]
	9.3	Comparative imagining of future outcomes	One motivation was to be able to sell products on the US market [22]	[22]

**Reward and Threat**<br><br>Definition: Techniques that involve providing incentives (material or social) or threats to encourage or deter specific behaviors.	10.1	Material incentive (behavior)	We provided Maasai individuals with tape measures and dosage charts to estimate dosage based on livestock body size; and thermometers to enable milk pasteurization [32]	[24,26,32,35,39,41,44]
	10.2	Material reward (behavior)	Participation in the AM reduction project was remunerated for farmers with 500.- Swiss Francs (~548.00 US Dollars) per farm per year [26]	[26,26,39,44]
	10.4	Social reward	Participants were not paid to participate; the only material incentives were free lunches at meetings and agricultural show tickets awarded to 5 farmers at the close of the project for significant changes to AMU [29]	[29]
	10.8	Incentive (outcome)	The only material incentives were free lunches at meetings and agricultural show tickets awarded to 5 farmers at the close of the project for significant changes to AMU [29]	[22,24,29]
	10.1	Reward (outcome)	Farmers received a yearly amount of 90 CHF (US $ 100.64) per livestock unit if they participated in the program [44]	(outcome)[29,44]
	10.11	Future punishment	Farmers with herds above the 80th percentile received a letter warning them that they were close to Yellow Card thresholds and Yellow Cards were issued from December 2010 onwards [27]	[27]
**Antecedents**<br><br>Definition: Behavioral change techniques that alter the surrounding conditions and situations to encourage individuals to adopt and sustain new behaviors.	12.1	Restructuring the physical environment	Farmers who participate have to keep cows in a free stall housing system as opposed to tie stalls [44]	[34,35,38,38,40,44]
	12.2	Restructuring the social environment	It allowed a pharmacy to divide large boxes that contain several packages into single packages of antimicrobials to sell smaller quantities of antimicrobials at the same price as did veterinarians [34]	[34]
	12.5	Adding objects to the environment	To promote pasteurization we provided thermometers [32]	[26,32,33,39]
**Scheduled Consequences**<br><br>Definition: Techniques that involve imposing behavior costs, applying punishments, or removing rewards for non-compliance with the intention of influencing certain behavior.	14.1	Behavior cost	If this target is not reached, a second-opinion veterinarian is involved to develop a strategy for reduction such as sampling and testing, vaccination strategies, management changes, etc. All costs are paid by the farmer [27]	[27]
	14.2	Punishment	If this target is not reached, a second-opinion veterinarian is involved to develop a strategy for reduction such as sampling and testing, vaccination strategies, management changes, etc. All costs are paid by the farmer [27]	[27]
	14.3	Remove reward	Finally it prohibited pharmacies and the pharmaceutical industry from offering economic incentives to veterinarians or others for the purpose of increasing product sales [34]	[34]
